# Antibiotics and Antibiotic Resistance—Mur Ligases as an Antibacterial Target

**DOI:** 10.3390/molecules28248076

**Published:** 2023-12-13

**Authors:** Vincent Hervin, Vincent Roy, Luigi A. Agrofoglio

**Affiliations:** ICOA UMR CNRS 7311, Université d’Orléans et CNRS, Rue de Chartres, 45067 Orléans, France; vincent.hervin@univ-orleans.fr

**Keywords:** peptidoglycan biosynthesis, Mur ligases, antibacterials, inhibitors

## Abstract

The emergence of Multidrug Resistance (MDR) strains of bacteria has accelerated the search for new antibacterials. The specific bacterial peptidoglycan biosynthetic pathway represents opportunities for the development of novel antibacterial agents. Among the enzymes involved, Mur ligases, described herein, and especially the amide ligases MurC-F are key targets for the discovery of multi-inhibitors, as they share common active sites and structural features.

## 1. Introduction

**Figure 1:**
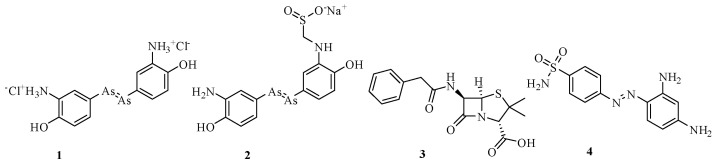
Structure of Salvarsan (**1**), Neosalvarsan (**2**), penicillin G (**3**), and sulfanilamide (**4**).

Microorganisms have existed on Earth for centuries, but it was not until the 17th century that Antoni van Leeuwenhoek, a renowned Dutch scientist, made groundbreaking observations identifying various microorganisms, including yeasts and even red blood cells [1]. His work marked an important moment in the history of biology, laying the foundation for microbiology and bacteriology. Following Leeuwenhoek’s discoveries, a series of events triggered a surge of interest in microbiology, leading to significant advancements in the field. In the years that followed, numerous scientists studied microorganisms, particularly those that caused major epidemics. In 1835, Agostino Bassi identified *Beauveria bassiana* as the microbial origin of the silkworm disease called muscardine [2]. In 1854, Filippo Pacini isolated and identified the *Vibrio cholerae* as a pathogen [3]; meanwhile, Casimir Davaine discovered the *anthrax bacillus* [4]. These discoveries propelled microbiology forward, driven by the efforts of figures like Robert Koch [5] and Louis Pasteur [6]. The early 20th century marked a significant period in the history of antibiotics. German scientist Paul Ehrlich created the first effective drugs (Figure 1) against syphilis in 1910 [7], and, later, Alexander Fleming made a groundbreaking discovery in 1928, observing that *Penicillium notatum* had the ability to inhibit the growth of *Staphylococcus aureus (S. aureus)*. This discovery [8] laid the foundation for the development of penicillin G **3** and other antibiotics such as sulfanilamide **4** [9,10].

**Figure 2:**
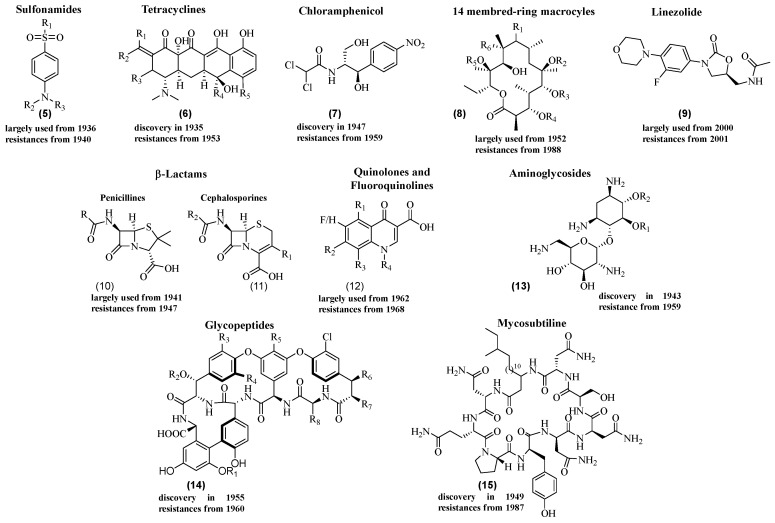
Some examples of families of antibiotics.

The golden age of antibiotic discovery occurred from the 1940s to the 1960s, with the identification of numerous families of antibiotics **5**–**15** (Figure 2) [11]. 

However, bacteria developed resistance mechanisms in response to antibiotic treatments [12,13]. This resistance is a significant global health threat, and the World Health Organization (WHO) considers it one of the most serious challenges to global health. Infections caused by resistant bacteria, coupled with diminishing antibiotic effectiveness, have raised alarms [14,15,16]. 

**Figure 3:**
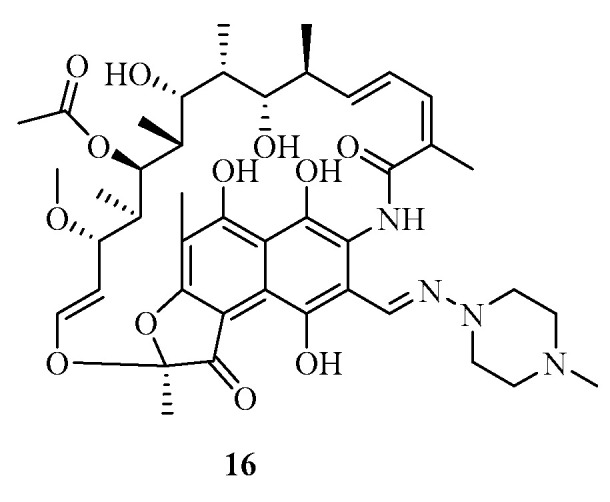
Structure of rifampicin (**16**).

Bacterial resistance can occur through different mechanisms [17,18,19,20,21,22,23,24,25,26,27], including extracellular resistance (biofilms), natural or innate resistance (inherent to specific strains), and acquired resistance (mutations or acquisition of resistance genes). Resistance mechanisms involve alterations in genes affecting interactions between bacteria and antibiotics, modifications of antibiotic targets, enzymatic mechanisms to prevent antibiotic binding, the hindrance of antibiotic entry through bacterial membranes, and the efflux of antibiotics from bacterial cells. This modification involves changes in the target’s genetic sequences, resulting in decreased efficacy of the antibiotic. For example, this phenomenon has been observed in a strain of *Mycobacterium leprae* resistant to rifampicin **16**, an antibiotic of the rifamycin family (Figure 3) [27].

**Scheme 1:**
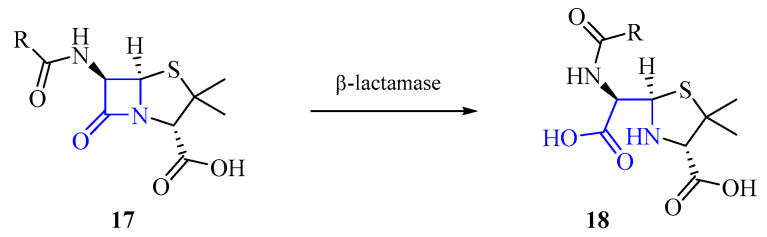
Hydrolysis by β-lactamase.

Enzymes produced by the bacteria can also inactivate the antibiotic by cleaving it. For example, enzymes known as “*β-lactamase*” are capable of hydrolyzing β-lactam rings found in antibiotics such as penicillins **17** (Scheme 1) to inactive compounds **18** [28]. β-lactams target bacterial wall biosynthesis by inhibiting the transpeptidase activity of penicillin-binding proteins (PBPs), which are involved in the final steps of peptidoglycan synthesis. The open form of β-lactams results in the loss of their biological activity against PBP transpeptidases.

The ability of an antibiotic to reach its target within bacteria can also occur via the bacterial membranes [29,30,31,32,33,34,35,36,37]. Despite the challenges of bacterial resistance, antibiotics remain effective against certain pathogenic bacteria. These antibiotics target specific bacterial processes, such as DNA replication, RNA polymerase activity, protein synthesis, and bacterial wall synthesis. For example, antibiotics can inhibit DNA gyrase and RNA polymerase [38,39,40,41,42], interfere with the metabolism of folic acid [43,44,45,46,47], disrupt protein synthesis [48,49,50,51,52,53], and target the bacterial wall synthesis process [54,55,56,57,58,59]. Efforts to combat resistance involve the development of new antibiotics targeting novel bacterial processes and exploring new therapeutic targets. These ongoing efforts are crucial in the fight against antibiotic-resistant bacteria and the preservation of effective treatment options. 

## 2. The Peptidoglycan Chain

**Figure 4:**
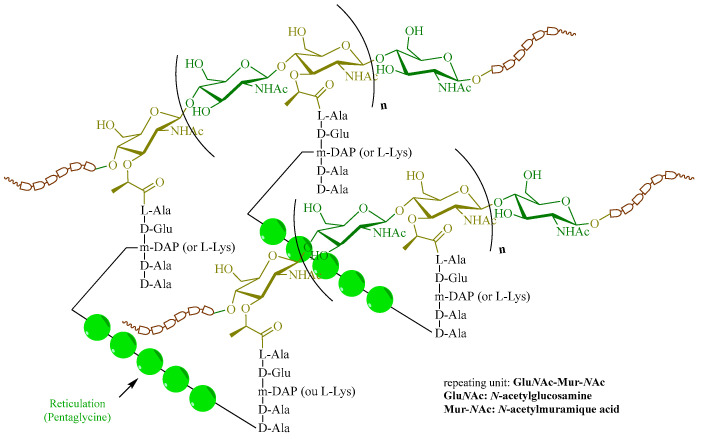
Peptidoglycan structures.

Peptidoglycan, also called murein, is a biopolymer present in all bacteria that provides protection from the external environment, especially osmotic pressure [60]. A notable difference between bacteria is that the cell wall is composed of about 95% peptidoglycan chains for Gram-positive bacteria and about 20% for Gram-negative bacteria, which explains the effectiveness of some antibiotics compared to others. Its complex chemical structure is defined by a cross-linked network in which a repeating unit contains an *N*-acetylglucosamine (NAG) and an *N*-acetylmuramic acid (NAM) linked by a β-1 bond → 4 (Figure 4) [61].

This peptidoglycan forms a three-dimensional network linked by cross-links simply composed of peptides, a pentaglycine here. This cross-linking is linked on both sides with two small peptides that are grafted by the carboxylic acid function of MurNAc. The chemical nature of these peptides differs according to the bacteria; in Gram-positive bacteria, the unnatural amino acid called *meso*-diaminopimelic acid (*m-DAP*) is found, whereas in Gram-negative bacteria, L-Lysine is present. This structure presents an impressive chemical diversity and explains the strength of the peptidoglycan chain in bacteria. Indeed, several elements that compose it testify to this, such as the MurNAc, a saccharide not present in eukaryotic cells, amino acids of the d series, and *m-DAP*. 

**Scheme 2:**
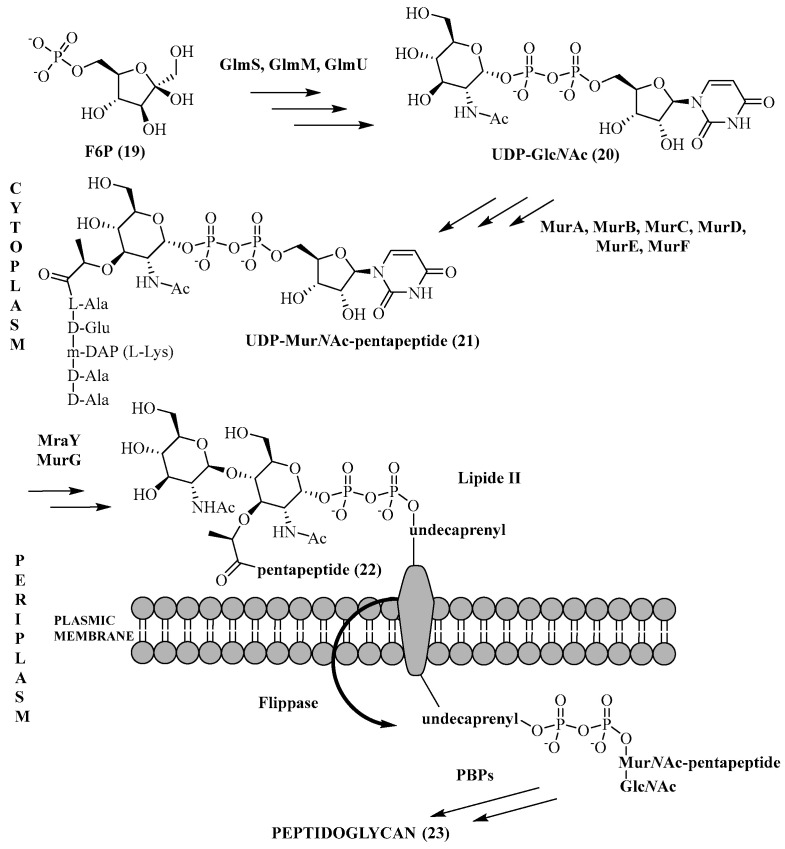
Key steps in peptidoglycan biosynthesis.

The biosynthesis of the peptidoglycan chain (Scheme 2) is a complex process that takes place in every part of the bacterium [62]. It is common to all Gram-positive or Gram-negative bacteria with some variations for *Mycobacterium tuberculosis* [63]. Each enzyme involved in this biosynthesis is important because the inhibition of one would lead to bacterial lysis and therefore cell death. During this biosynthesis, the formation of several key intermediates is obtained in each site of the bacterium (cytoplasm, membrane, and periplasm). The starting point of this synthesis is the conversion of fructose-6-phosphate (F6P, **19**) to uridine diphosphate-*N*-acetylglucosamine (UDP-GlcNAc, **20**). Then, a lactoyl function is introduced on this substrate, followed by a pentapeptide chain to form uridine diphosphate *N*-acetylmuramoyl-pentapeptide (UDP-MurNAc-pentapeptide, **21**). This entity, which is nothing but a part of the peptidoglycan-repeating unit, binds to undecaprenylphosphate (C55P) of the plasma membrane and is then glycosylated to obtain Lipid II (**22**). An enzyme called flippase will allow Lipid II to pass from the internal face of the membrane to its external face [64]. Finally, this monomer is polymerized in the periplasm by transglycosylation and transpeptidation steps to end up with the mature peptidoglycan (**23**). 

**Figure 5:**
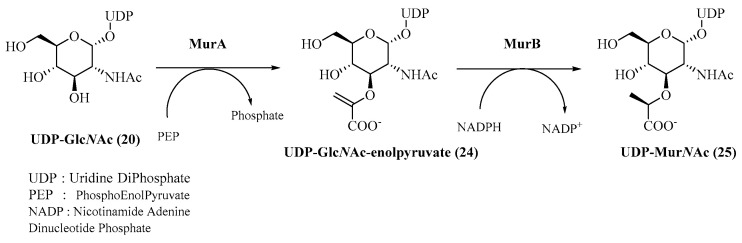
MurA- and MurB-catalyzed formation of UDP-MurNAc.

Mur ligases are involved at several parts of this process; their role is to catalyze the formation of a peptide bond with the corresponding amino acid on the substrate, which is specific to each of these enzymes. These enzymes have all been identified as non-ribosomal ATP-dependent proteins in the cytoplasm, and MurA and MurB allow the biosynthesis of UDP-MurNAc (**25**) from UDP-GlcNAc (**28**) (Figure 5).

**Scheme 3:**
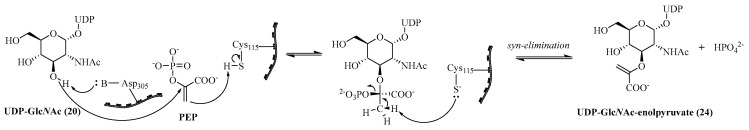
Mechanism of MurA−catalyzed UDP-MurNAc enolpyruvate (adapted from Ref. [47]).

MurA or UDP-GlcNAc enolpyruvyltransferase catalyzes the transfer of an enolpyruvate moiety from phosphoenol pyruvate (PEP) to UDP-GlcNAc (**20**) with phosphate release. The crystallographic structure [65] of MurA as well as its mechanism [66] have been well identified (Scheme 3). Its mode of action involves an addition–elimination mechanism: the *anti-*addition of PEP on UDP-GlcNAc (**20**) is catalyzed by Asp_305_ (numbering of MurA from *Escherichia coli*) and Cys_115_ of MurA to form the corresponding tetrahedral intermediate, and then the *syn-elimination* allows UDP-GlcNAc enolpyruvate (**24**) to be obtained.

**Figure 6:**
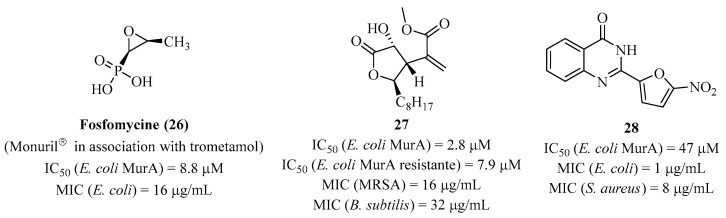
Structure of fosfomycin (**26**) and compounds **27** and **28**.

Currently, the only antibiotic used that specifically targets MurA is fosfomycin (**26**) (Figure 6). It is a PEP analog which binds irreversibly to Cys_115_ (numbering of MurA from *Escherichia coli)* of the active site of MurA, rendering the enzyme inactive and causing cell death. However, there are many mechanisms of resistance to this compound: mutations of the enzyme, cellular permeability, or an enzymatic action that inactivates the antibiotic. Similarly, innate resistance of certain species such as *Mycobacterium tuberculosis* or *Chlamydia trachomatis* exist where an Asp [67] replaces the targeted Cys residue. Numerous covalent and noncovalent inhibitors have been developed [68]. Avenaciolide compounds [69] (isolated from *Neosartorya fischeri*) are tetrahedral intermediate inhibitors, while quinazolinone analogs [70] are competitive inhibitors. Compound **27** is promising because it shows good biological activities with an MIC of 16 μg/mL^−1^ and 32 μg/mL^−1^ on methicillin-resistant *Staphylococcus aureus* (MRSA) and *Bacillus subtilis (B. subtilis),* respectively. Furthermore, it was shown to be specific for MurA enzymes with an IC_50_ of 2.8 μM on MurA from *Escherichia coli (E. coli)* and 7.9 μM on resistant MurA. Compound **28** is active on *Escherichia coli* and *Staphylococcus aureus,* respectively, with an MIC of 1 μg/mL^−1^ and 8 μg/mL^−1^ while showing an IC_50_ on MurA of 47 μM.

**Scheme 4:**
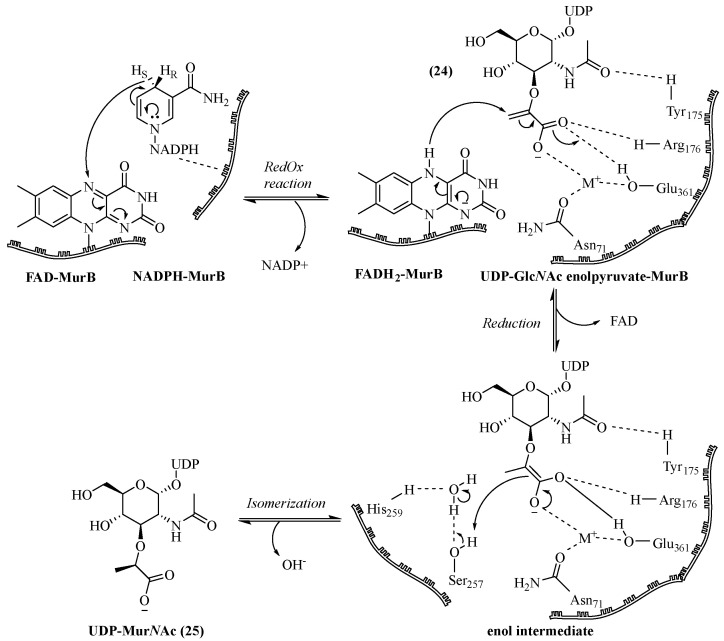
MurB-catalyzed mechanism of UDP-MurNAc formation (adapted from ref. [72]).

MurB or UDP-GlcNAc enolpyruvate reductase catalyzes the stereoselective reduction of UDP-GlcNAc enolpyruvate (**24**) to UDP-MurNAc (**25**) with the action of a co-enzyme called Nicotinamide Adenine Dinucleotide Phosphate (NADPH) [71]. This enzyme also contains an active site with the Flavin Adenine Dinucleotide cofactor (FAD). The crystallographic structure [72,73] of MurB and its mechanism [74,75] have been elucidated (Scheme 4). The first step of the mechanism involves the formation of the FAD-MurB complex, which acts as a redox intermediate. Similarly, after the formation of the second NADPH-MurB complex, the reduction of FAD by NADPH leads to the reaction intermediate FADH_2_ -MurB by transferring the H4 (*pro-S)* from NADPH to the N of FAD. After the release of NADP+, UDP-GlcNAc enolpyruvate (**24**) binds to the enzyme. The second step is the reduction of the latter by the FADH_2_ -MurB thanks to the transfer of hydrogen in C-3 on the enolpyruvate part. After the release of FAD, the enolate intermediate obtained is stabilized by the carboxylic acid of the substrate with the enzyme. Finally, the isomerization of the substrate to the final product UDP-MurNAc (**25**) requires the presence of water.

**Figure 7:**
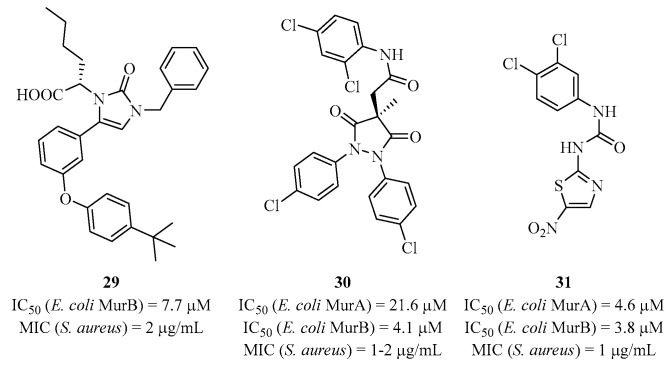
Structure of some MurB inhibitors.

Currently, there is no antibiotic used for the MurB of bacteria. However, few inhibitors have been developed (Figure 7) such as the imidazolinone **29**, one of the first inhibitors targeting the MurB substrate site from *Escherichia coli*. It shows activity against *Staphylococcus aureus* strains [76]. Compounds **30** and **31** have been identified as multi-inhibitors of MurA/MurB targeting FAD, with interesting activities on *Staphylococcus aureus* strains [77,78].

**Scheme 5:**
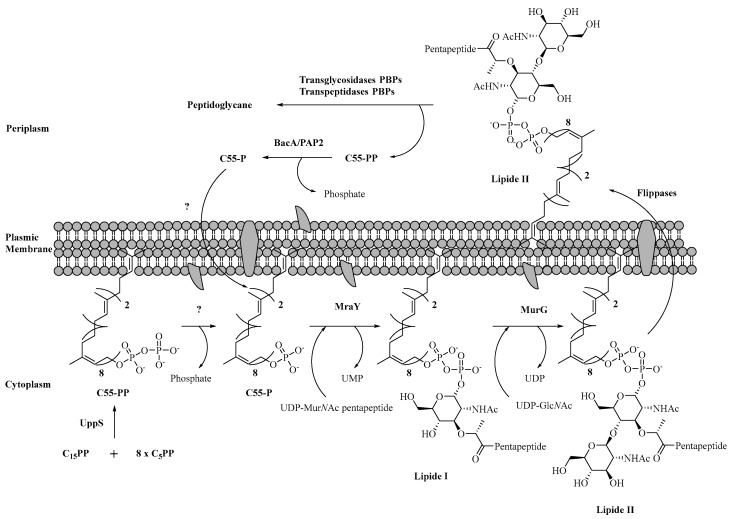
Enzymatic synthesis of Lipid II and its translocation.

Finally, the last cytoplasmic steps allowing the formation of UDP-MurNAc pentapeptide from the UDP-MurNAc (**25**) involve MurC-F, which are grouped into a family of four amino acid ligase enzymes that form a peptide bond in the presence of the corresponding amino acid [79,80]. Other enzymes at the plasma membrane are responsible for the continuation of this synthesis (Scheme 5). Among them, MurG, a glycosyltransferase, catalyzes the glycosylation between Lipid I and a GlcNAc unit from UDP-GlcNAc (**20**) to form Lipid II. This essential enzyme is highly conserved in all bacterial species. However, glycosyltransferases are present in a large majority of cells in both prokaryotes and eukaryotes, which requires a high specificity of potential MurG inhibitors in drug design [81]. 

**Figure 8:**
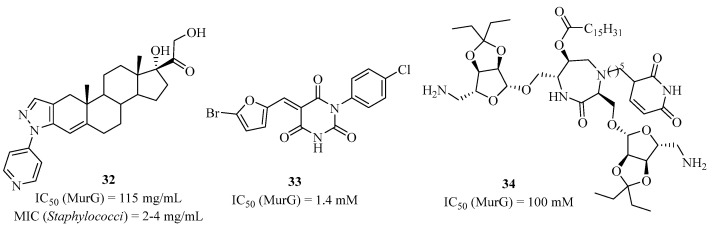
Selected MurG inhibitors.

The crystallographic structure of MurG has been solved, as well as its mechanism, by Walker et al. [82,83,84]. Other teams have shown that the structure of MurG is able to interact with other proteins such as MraY [85], and with the MurE-MurF dimer complex [86], which allows the substrate to evolve with small distances within this protein–protein complex. Several inhibitors targeting MurG have been developed as the pentacyclic compound (**32**) (Figure 8) [87]. In addition, the use of high-throughput screening has allowed the development of new inhibitors against the MurG, such as the pyrimidinetrione (**33**) [88,89]; more recently, the diazepanone analog (**34**) was found to inhibit both MraY and MurG [90].

## 3. MurC-F Ligases as New Antibiotic Targets

In the fight against bacterial resistance, another effective way is to go after seldom exploited targets that do not show any resistance mechanism. In this context, enzymes involved in bacterial wall biosynthesis meet this criterion. Among some of these enzymes, resistances have already appeared, such as in the periplasm where penicillin that targets PBPs has become ineffective with the appearance of β-lactamases or with glycopeptides that target Lipid II and has become less active. By contrast, Mur ligases have an activity further upstream in the biosynthesis. These enzymes have many advantages that make them an innovative therapeutic target: they are essential for bacterial survival, ubiquitous within prokaryotes with no equivalent in eukaryotes. To date, they are relevant targets in the development of multi-inhibitors and they do not present any resistance mechanism [91]. 

**Scheme 6:**
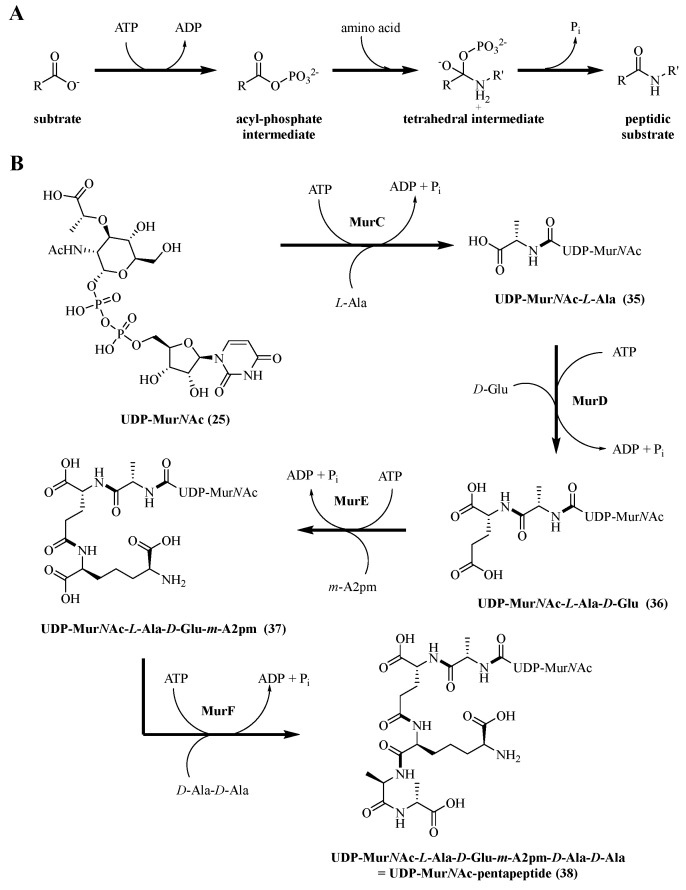
(**A**) General mechanism; (**B**) general scheme of UDP-MurNAc-pentapeptide formation.

MurC-F ligases are responsible for the formation of the Lipid I precursor (UDP-MurNAc-pentapeptide, **38**) from UDP-MurNAc (**25**) by the successive synthesis of tri-, tetra-, and penta-peptides by MurC, MurD, MurE, and MurF (Scheme 6) [92]. The characterization of the protein sequences of MurC-F enzymes has been performed on different bacterial strains, both Gram-positive and Gram-negative [93]. Even if sequential differences between species can be observed, each of them shares the same structural topology, i.e., a protein divided into three different domains with a conserved active site [94]. The main differences in the protein sequences of the Mur ligases are that the amino acid binding site differs depending on which one is used. The MurC enzyme catalyzes the addition of the first amino acid of the peptide chain bound to the UDP-MurNAc (**25**). In most species, this amino acid is L-Ala, but in very rare cases, other amino acids such as glycine or L-Ser are used. MurD catalyzes the addition of the second amino acid to the substrate. Except for some variations in amino acids due to modifications in biosynthesis, the amino acid is a D-Glu in all species. Studies have shown the importance of having the D-enantiomer of glutamic acid because L-Glu is not a substrate for MurD. For MurE, which catalyzes the third amino acid addition, the amino acid is either a *meso-diaminopimelic* acid (*m-A2pm*) in most Gram-negative bacteria and in *Bacilli* species, while in Gram-positive bacteria, the amino acid is an L-Lys. Studies have shown that MurE contains a very specific active site for its own amino acid; if the wrong amino acid were to be incorporated, it would result in cell lysis. Finally, MurF catalyzes the addition of an unnatural dipeptide D-Ala-D-Ala. In some resistant bacterial species, a modification of this dipeptide by D-Ala-D-Ser or by D-Ala-D-Lac can be observed. To date, each of these enzymes has been purified and studied with the corresponding substrates and co-substrates [95]. This allowed the authors to propose the active sites of the Mur ligases as well as the reaction mechanism where we present these parameters.

The stability parameters of Mur ligases are important to determine the specificity and kinetic parameters. A characterization study of these parameters was performed on MurC-F enzymes from *Mycobacterium tuberculosis* [96]. The specific activities of MurC-F are calculated by measuring the concentration of the released inorganic phosphate (Scheme 6B). They are estimated to be 1.2, 0.8, 1.3, and 0.9 μmol/min/mg/protein (μmol of phosphate formed per minute per milligram of enzyme) for MurC, MurD, MurE, and MurF, respectively. The stability of each of the proteins showed a great sensitivity according to the temperature and the pH of the medium. Indeed, the enzymatic activity remains stable under 40 °C with an optimal activity between 35 °C and 40 °C, while a higher temperature (between 45 and 70 °C) shows a significant decrease in the activity. Moreover, enzymes are sensitive to the pH of the buffered medium since the optimal pH is estimated between pH 8 and 8.5. Small variations with a more acidic or basic pH are enough to strongly decrease the activity. Mur ligases use the energy of the phosphate bond to catalyze the formation of the amino acid amide bond on the growing peptide chain (Scheme 6). This mechanism, common to all four Mur ligases, starts with the elaboration of a complex with the enzyme and its substrates in the following way: first, the ATP binds to the enzyme, and then the substrate UDP sugar is added, as well as the amino acid at the end. In the active site, the first step is to activate the carboxylic acid of the substrate UDP sugar with ATP (Scheme 6A) to form the acyl phosphate intermediate. Two Mg ions^2+^ will create a bridge between the negatively charged groups of ATP and the substrate UDP sugar to facilitate the phosphorylation of the carboxylate group of the substrate. Then, the second step involves the amino acid, which displaces the phosphate by a nucleophilic attack with the formation of the tetrahedral intermediate. Finally, the release of the phosphate P_i_ allows for obtaining the peptide substrate. The catalytic activity of a base is necessary to recover the proton from the amine group. Other studies have shown that the ATP phosphate could replace this base or the enzyme itself since this would better fit the normal energy scale of the reaction [97]. With MurC-F, therefore, L-Ala, D-Glu, m-A2pm, and the dipeptide D-Ala-D-Ala can be introduced successively in the presence of ATP (Scheme 6B).

In addition to the mechanistic data of these enzymes, X-ray structures of MurC-F ligases for several different species have been elucidated [98,99,100,101].

Inhibitors of MurC-F *Escherichia coli* were developed since they have the advantage of containing multiple active sites per enzyme, allowing researchers to target specific areas of interest to design molecules that may have biological activity benefits. Moreover, the increasing emergence of bacterial resistance challenges the conventional approach to developing antibacterials. Mur ligases are ideal targets for this purpose since they share similar three-dimensional structures, making them a set of targets for a single compound. 

### 3.1. Inhibitors from “Medicinal Chemistry Approach”

This follows a simple mono-therapeutic model aimed at designing a drug for a specific target in a particular region or event. This approach has proven effective in the development of numerous antibiotics. The identification of potential hits (molecules of interest) involves in silico, in vitro, and in vivo studies [102]. The first characterized inhibitors targeted domain 3 of Mur ligases and emerged in the 1990s, along with the initial elucidation of crystallographic structures.

#### 3.1.1. Amino Acid Mimics of Mur Ligases

**Figure 9:**
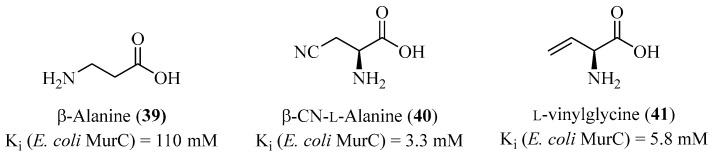
Structure of L-Alanine analogs.

The exploration of MurC inhibition using analogs of L-Alanine began early, even before the complete structure of the enzyme was known. Takahashi et al. showed that glycine could inhibit the addition of L-Alanine to the UDP-MurNAc substrate as a competitive inhibitor [103]. Several studies a few years later, including those by Parquet [104] et al. and VillaFranca et al. [105], demonstrated that various L-amino acids, without incorporation into the natural substrate, could inhibit MurC. D-configurations of amino acids, however, showed no inhibitory activity. For example, the three following amino acid analogs, **39**, **40**, and **41** (Figure 9), were the most potent competitive inhibitors, ranging from millimolar to minimum activity for **76**.

**Figure 10:**
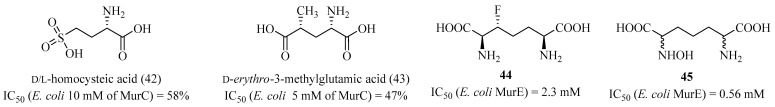
Structure of D-glutamic acid analogs and of meso-diaminopimelic acid analogs.

Inhibitors of MurD using analogs of D- or L-glutamic acid showed similar results as those for MurC. Van Heijenoort et al. demonstrated weakly active analogs, as seen with compounds **42** and **43** with residual activities of 58% and 47%, respectively, at a concentration of 10 mM (Figure 10) [106].

This same team conducted similar studies on analogs of meso-diaminopimelic acid on the MurE enzyme [107]. Among synthetized products, compounds **44** and **45** were the best analogs in terms of MurE inhibition, although their activity was weak, with IC_50_ values of 2.3 mM and 0.56 mM for **44** and **45**, respectively. For MurF, the literature does not show results containing derivatives with motifs from the D-Ala-D-Ala dipeptide.

#### 3.1.2. Heterocyclic Inhibitors

**Figure 11:**
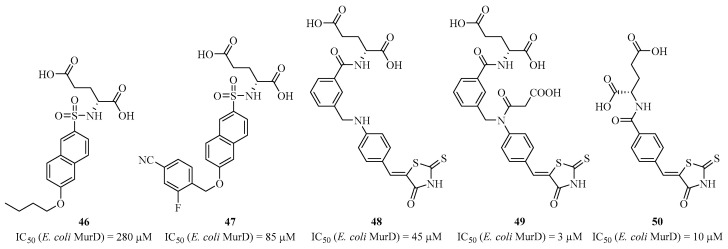
Structure of sulfonamide and heterocyclic analogs of D-glutamic acid.

In 2001, Gobec et al. based their work on this mechanism with MurD and the tetrahedral transition state to design phosphino-alanine derivatives, but without significant inhibition [108]. In 2007, the same group published small compounds, such as sulfonamides of D-glutamic acid. They co-crystallized inhibitors like compound **46** (Figure 11) with the MurD enzyme to better understand its binding site [109]. This allowed the group to develop other analogs with improved activity and the ability to crystallize the compounds, such as compound **47** with an IC_50_ of 85 μM [110]. The modification of position 6 of the naphthalene in **46** allowed the crystallographic structure of the inhibitor/MurD complex to show that this position enables interactions between the substituents and the di-phosphate binding site of domain 1. Subsequently, Gobec et al., in collaboration with Mašič’s group, performed ligand-docking studies, leading to the identification of a new interesting structure that could bind to the di-phosphate binding site. These structures **48**–**50** contained rhodanine-type heterocycles, such as compounds, which was active with an IC_50_ of 45 μM (for **48**).

**Figure 12:**
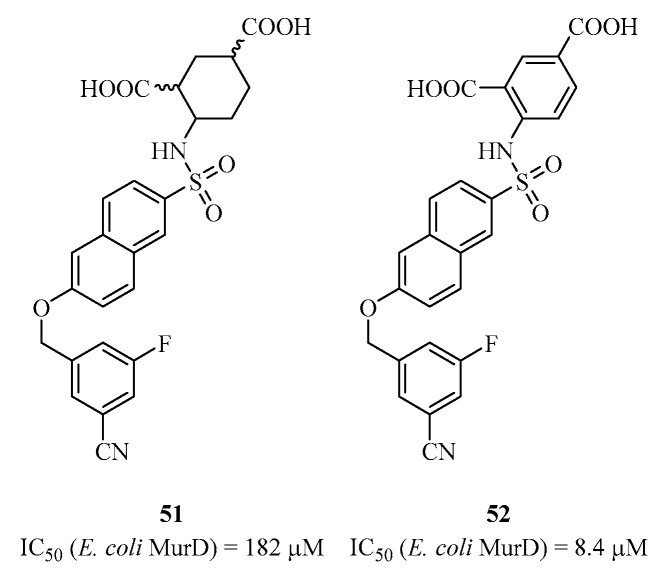
Structure of analogs **51** and **52** targeting MurD.

Subsequent SAR studies resulted in the discovery of analogs with improved biological activities, such as compounds **49** and **50** with IC_50_ values in the micromolar range [111,112,113]. These recent studies enabled the synthesis of a large library of hybrid molecules capable of binding both to the active site of the amino acid in domain 3 and to the di-phosphate pocket in domain 1, using in silico analysis and considering that the D-glutamic acid part must remain. However, no molecule with significant in vivo bacterial activity has been identified. Additionally, some synthesized compounds, such as those containing the “ene-rhodanines” heterocycle, may be considered as PAINS (pan-assay interference compounds) that produce false positives in the biological experimentation used to determine IC_50_ [114]. MurC presents a unique case where Lee et al. synthesized derivatives of benzylidene rhodanines [115]. Gobec et al. attempted to develop sulfonamide inhibitors of D-glutamic acid targeting MurE [116]. Subsequently, they synthesized a second generation of non-D-glutamic acid sulfonamide inhibitors that mimic it with rigid diacids. These inhibitors were found to be active on MurD, not MurE [117]. Compounds **51** and **52** (Figure 12), exhibiting IC_50_ values of 182 μM and 8.4 μM, respectively, were co-crystallized with the enzyme, and the structures showed that these inhibitors bind at the D-glutamic acid position, where the 2-cyano-4-fluoro-phenyl occupies the uracil pocket.

### 3.2. Inhibitors Mimicking the Natural Substrate UDP-MurNAc-(Peptide)

#### 3.2.1. Phosphinic Inhibitors

**Figure 13:**
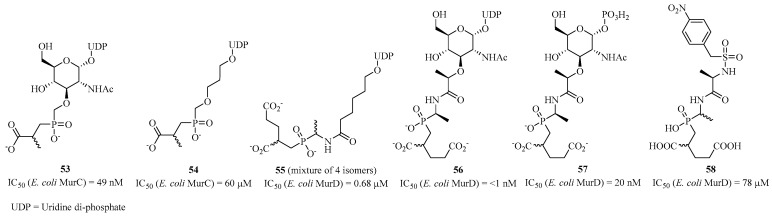
Structure of phosphinic analogs of MurC and MurD.

One of the first families of inhibitors is the “phosphinic” compounds that target the natural substrate-binding site in domain 1 of Mur ligases. The phosphinic acid in these molecules provides a tetrahedral geometry that blocks the enzymatic mechanism, and the uridine part of the compounds allows them to position themselves in the active site [118,119]. For MurC, AstraZeneca published the synthesis of two highly active inhibitors, **53** and **54**, with respective IC_50_ values of 49 nM and 60 μM (Figure 13) [120]. Compound **53** forms a stable enzyme/ATP/inhibitor and enzyme/ADP/inhibitor complex, which stops the enzymatic activity [121]. The first phosphinic inhibitor 55 was synthesized for MurD in 1996 by Blanot et al., which was active with an IC_50_ in the micromolar range [122]. Similarly, Merck published other inhibitors in 1998 with similar structures. They showed that adding the glucosamine part to compound **56** improved its activity, reaching IC_50_ values in the nanomolar range [123]. Later, Blanot et al., in collaboration with Gobec’s team, published simplified inhibitors by retaining only the peptide-phosphinic part, based on compound **57**, with an IC_50_ of 20 nM [124]. Even without improving this inhibitory activity, they still published active compounds like compound **58**, which was the most active with an IC_50_ of 78 μM.

**Figure 14:**
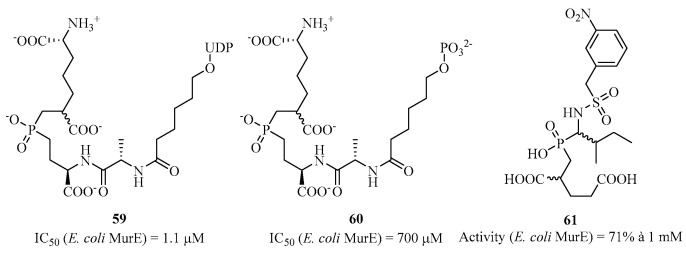
Structure of phosphinic analogs of MurE.

For MurE, Merck’s group synthesized compounds **59** and **60** with respective IC_50_ values of 1.1 μM and 700 μM (Figure 14), similar to compound **56** for MurC [125]. Similarly, Gobec et al. used **60** as a starting point to add various substituents, but without significant inhibition, with compound **61** showing the best residual activity at 71% at 1 mM [126]. Docking studies of an analog of **61** showed that this family of molecules is not transition-state inhibitors but rather competitive inhibitors of the substrate.

Besides the good enzymatic activities of these phosphinic analogs, none have shown antibacterial activity, which could be explained by their low cell wall penetration. However, an important point to consider in the design of these derivatives is that the uridine part is beneficial for retaining good IC_50_ values for each of the Mur ligases. Moreover, the total synthesis of phosphinic compounds proves to be challenging in terms of the number of steps. Several research groups have explored the laboratory synthesis of various natural substrates of Mur ligases, which has served as a synthetic tool, knowing that the nucleosidic synthesis of di-phosphate derivatives is a true challenge [127,128,129,130,131].

#### 3.2.2. Peptidic Inhibitors

**Figure 15:**
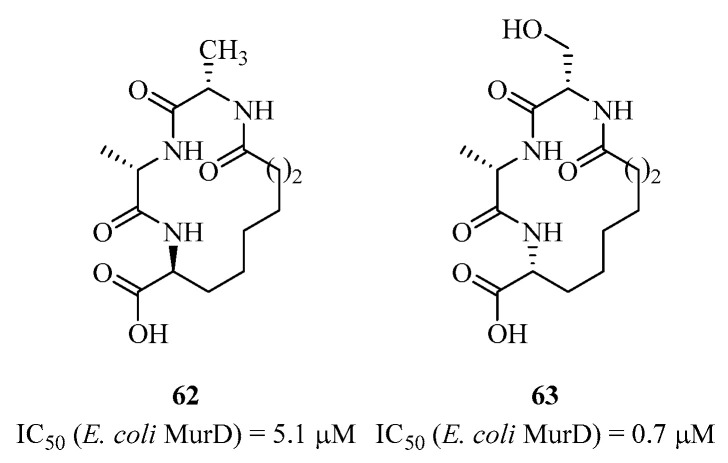
Structure of peptides **62** and **63** targeting MurD.

In 2003, Fishwick et al. published small peptides as analogs of the UDP sugar substrate targeting MurD [132]. This peptidic macrocycle **62** was designed based on the enzyme’s crystallographic structure and the docking of this potential inhibitor in the active site. Several analogs were synthesized, with compound **63** showing the best activity with an IC_50_ of 0.7 μM (5.1 μM for **60**) (Figure 15). There are also a few examples where authors developed peptidic derivatives targeting MurE [133,134]. 

On the other hand, several groups of biologists have been interested in peptide synthesis using the phage display technique with different peptide libraries. Several studies have identified peptides targeting MurC [135], MurD [136], MurE [137,138], and MurF [139], inhibiting with weak IC_50_ values in the mM range.

#### 3.2.3. Heterocyclic Inhibitors

**Figure 16:**
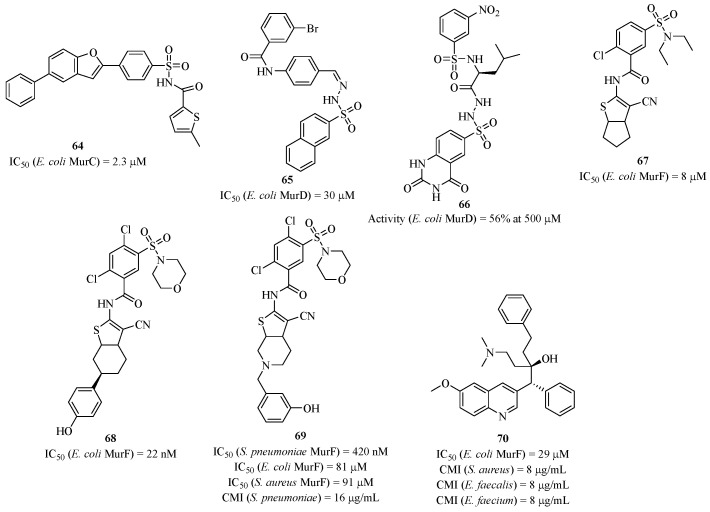
Structure of compound **64** targeting MurC, compounds **65** and **66** targeting MurD, and compounds **67**–**70** targeting MurF.

A very rapid technique to obtain inhibitors is to screen molecules through biological assays. AstraZeneca did this by screening its compound library targeting MurC [140]. Compound **64** was identified as an inhibitor with an IC_50_ of 2.3 μM (Figure 16). Its action seems to target the di-phosphate binding site, but the mode of inhibition has not been clearly elucidated. Moreover, it shows binding affinities to other proteins, such as bovine serum albumin. For MurD, Obreza’s team synthesized sulfono-hydrazine derivatives that also mimic the UDP sugar substrate’s di-phosphates [141]. Compound **65** showed the best IC_50_ of 30 μM but did not exhibit antibacterial activity. Additionally, the same team developed other similar analogs, like compound **66**, but without improving its activity [142]. For the same enzyme, Gobec’s team performed virtual screening of a molecule library, which led to the identification of inhibitors [143].

A research group from Abbott Laboratories conducted a high-throughput affinity screening of a molecule library targeting MurF, which highlighted molecule **67** as an attractive candidate [144,145]. Enzymatic studies revealed an IC_50_ of 8 μM, and through SAR studies, this result was optimized with compound **68**, which had an IC_50_ of 22 nM. Compound **67** was co-crystallized with MurF and bound to the active site in place of the natural UDP sugar substrate in a conformation called “closed” of the enzyme [146,147]. However, these compounds did not exhibit antibacterial activity, even in the presence of permeable strains, which may indicate non-specific interactions with other proteins. Based on these results, Gobec’s team decided to take up the structural motif of compound **68** to improve its antibacterial activities [148]. This led to compound **69**, which was active against *Staphylococcus pneumoniae* strains with an MIC of 16 μg/mL. However, **69** and its analogs did not exhibit activity against other bacterial strains. The authors published a second generation of these compounds active against several strains [149], where their antibacterial action resulted from membrane degradation. Other heterocyclic analogs were synthesized by Gobec’s laboratory with a few antibacterial activities [150,151]. In 2006, a research group from Johnson & Johnson developed pyrimidine derivatives that inhibited MurF but showed no in vivo activity [152]. Only from 2007 did the same group identify, through molecule screening, hydroxyquinoline-type derivatives of interest, such as compound **70** with an IC_50_ of 29 μM. Although this compound exhibited activity against both Gram-positive and Gram-negative bacterial strains, it is possible that compound **70** interacted with other proteins other than MurF [153,154].

### 3.3. Inhibitors Mimicking the Co-Substrate ATP

**Figure 17:**
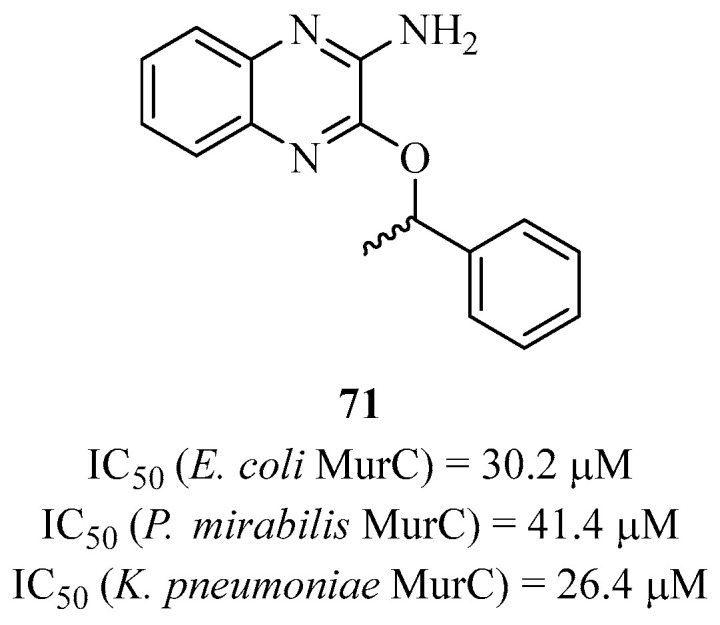
Structure of compound **71** targeting MurC.

There are very few inhibitors of this type in the literature targeting Mur ligases. This can be explained by the fact that there are many ATP-dependent enzymes, including kinases, resulting in a very severe lack of selectivity. Therefore, the few synthesized inhibitors were obtained through screening techniques of molecule libraries. In 2008, Dougherty’s team from the pharmaceutical group Pfizer carried out a screening of its molecule library and managed to demonstrate that compound **71** is a competitive inhibitor of ATP, selectively targeting MurC from only a few enterobacterial strains closely related to *Escherichia coli* (Figure 17). However, this compound did not show antibacterial activity [155].

### 3.4. Natural Inhibitors

**Figure 18:**
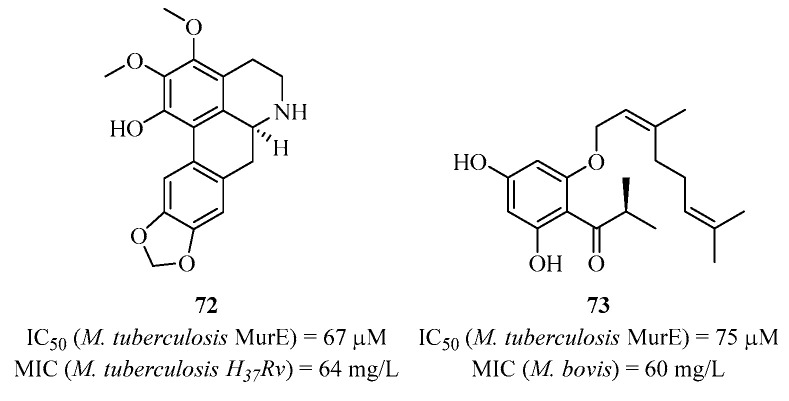
Structure of natural compounds **72** and **73** targeting MurE.

Using the same molecule screening technique (in this case, from plants), a research group identified natural compounds like **72** and **73** that were found to be active against MurE (Figure 18) [156,157,158]. Compound **72** showed an IC_50_ of 67 μM against MurE from *M. tuberculosis* and weakly inhibited the growth of a mutant strain of *Mycobacterium tuberculosis*. As for compound **73**, it had an IC_50_ of 75 μM against MurE and was active against a panel of Gram-positive and Gram-negative strains. It also acted on efflux by inhibiting a protein responsible for NorA.

### 3.5. Multi-Target Synthetic Approaches

The synthesis of multi-inhibitor compounds involves a “multi-therapeutic” model, where a molecule is produced to inhibit multiple biological processes by targeting several enzymes simultaneously. Generally, the choice is made on similar enzymes with close activities, as demonstrated with the Mur ligases. The reason behind this concept is to combat bacterial resistance to the purported new antibacterial compound built on these criteria. The advantage of multi-inhibition is that it leaves no chance for bacteria to react or adapt when exposed to this toxic agent. In the literature, there are a few relatively recent examples of multi-targeted Mur ligases that we present. This principle poses a challenge in developing such compounds while retaining attractive biological properties.

#### 3.5.1. Mimicking the Amino Acid of Mur Ligases

**Figure 19:**
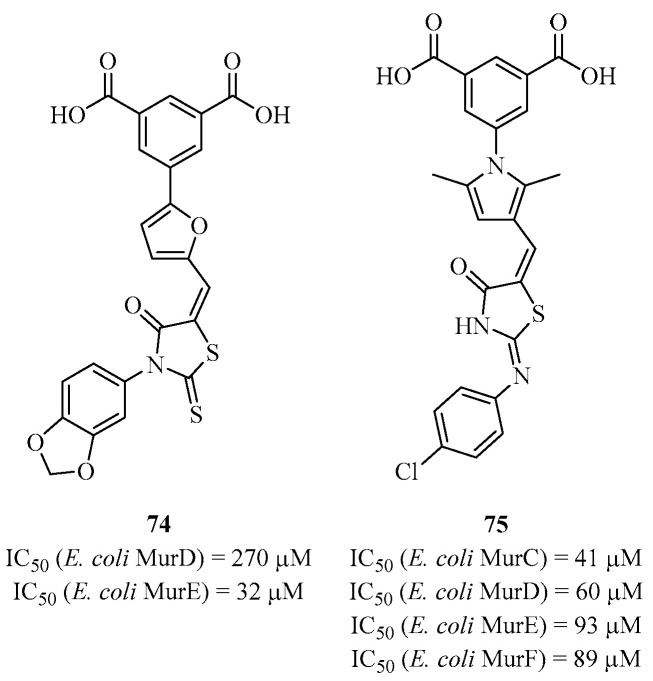
Structure of multi-inhibitors **74** and **75** targeting MurC-F.

Solmajer et al. and Gobec et al. developed new structures derived from D-glutamic acid using several in silico approaches, as they had done for classical inhibitors. Among these previous studies, they also identified multi-active compounds, and SAR studies were carried out based on these results [159]. The 1,3-phenyl-dicarboxylic acid, which is the cyclic mimic of D-glutamic acid, seems to play an important role in the binding to each of the Mur ligases. Initially, the team developed analogs of this type targeting MurD and MurE, as shown by compound **74** with an IC_50_ of 270 μM on MurD and an IC_50_ of 32 μM on MurE [160]. Subsequently, the group developed other analogs by modifying the heterocyclic part that fits into the di-phosphate pocket while retaining the 1,3-phenyl-dicarboxylic acid [161]. They synthesized compound **75**, which is the best in the series and capable of inhibiting all four Mur ligases with IC_50_ values of 41 μM, 60 μM, 93 μM, and 89 μM for MurC, MurD, MurE, and MurF, respectively (Figure 19). Although these molecules are multi-active, with compound **75** being one of the best multi-inhibitors synthesized to date, there is no revealed antibacterial activity. The group attempted to measure activities on different strains with analogs close to **75** (without the di-carboxylic acid groups), but the results only showed weak activities. 

#### 3.5.2. Mimicking the Natural Substrate UDP-MurNAc(-Peptide)

##### Phosphonic Acid Inhibitors

**Figure 20:**
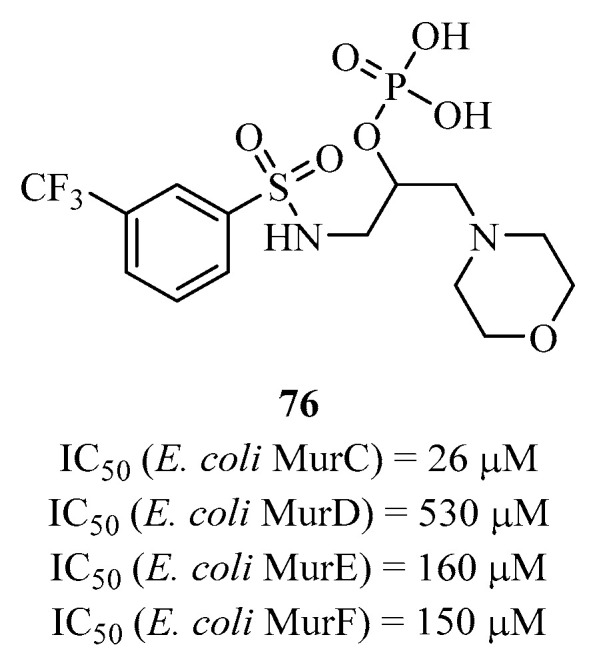
Structure of multi-inhibitor **76** targeting MurC-F.

Gobec et al. developed a series of phosphoric acid analogs of hydroxyethylamines as a bioisostere of the tetrahedral intermediate of the UDP sugar substrate [162]. Compound **76** is the best analog in this series, active against all four enzymes with IC_50_ values of 26 μM, 530 μM, 160 μM, and 150 μM for MurC, MurD, MurE, and MurF, respectively (Figure 20). However, these compounds do not show antibacterial activities. These analogs, including **76**, are the only phosphorylated derivatives that are multi-active against Mur ligases. 

##### Heterocyclic Inhibitors

**Figure 21:**
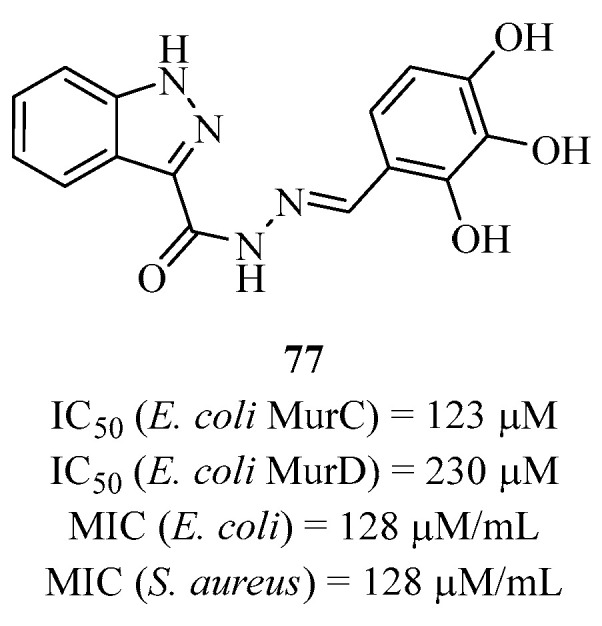
Structure of multi-inhibitor **77** targeting MurC and D.

After observing that the *N*-acyl hydrazone structure has potential in terms of biological activity in medicinal chemistry, Gobec’s group designed analogs around this structure and showed that some are active, like compound **77** with IC_50_ values of 123 μM and 230 μM for MurC and MurD, respectively (Figure 21) [163]. Moreover, this compound showed weak antibacterial activities against *Escherichia coli* and *Staphylococcus aureus*. The group also designed other heterocyclic structures to adopt a more closed conformation than with the natural substrates of enzymes MurD and MurF but still show activities on different strains.

**Figure 22:**
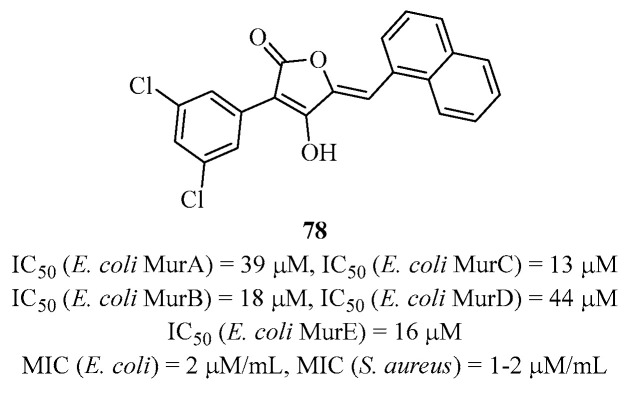
Structure of multi-inhibitor **78** targeting MurA-F.

Gobec et al., who synthesized a good number of benzylidenesulfonyl hydrazine analogs, showed that some of them were multi-inhibitors [164]. They performed SAR studies to optimize enzymatic activities but without antibacterial activities. Singh’s laboratory identified several multi-active structures of Mur ligases through different screenings, including pulvinones [165] and phenyl dihydrothienopyrazolol [166] with activities against multiple strains. Recently, they identified a naphthyl-type tetronic acid structure capable of inhibiting not only all four Mur ligases but also MurA and MurB [167]. Compound **78** showed the best activities in the series with an IC_50_ in the range of 20 μM for each enzyme, and it inhibited the bacterial growth of *Escherichia coli* and *Staphylococcus aureus* strains (Figure 22).

#### 3.5.3. Mimicking the Co-Substrate ATP

**Figure 23:**
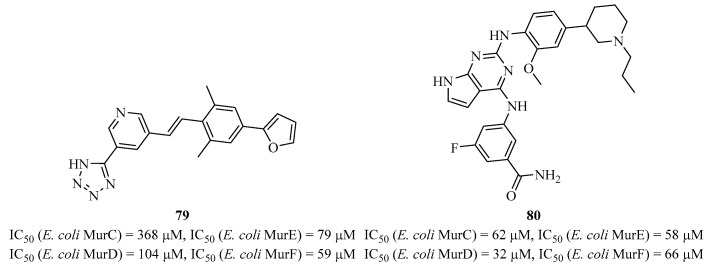
Structure of multi-inhibitor compounds **79** and **80** targeting MurC-F.

Very recently, Zega et al. identified several compounds with different structures that exhibit multi-inhibitory activities against MurC to MurF. These compounds were identified through screening a collection of molecules originally designed to inhibit kinases by the competitive inhibition of ATP [168]. Compounds **79** and **80** are multi-inhibitors of MurC-F with IC_50_ values ranging from 10 to 368 μM (Figure 23). Because these compounds mimic ATP, the authors conducted kinetic and NMR studies on compound **79** and showed that it actually binds to the amino acid binding site. They also performed additional biological analyses with eukaryotic kinases and demonstrated that these molecules are specific and promising for the development of new antibacterials.

#### 3.5.4. Natural Analogs

**Figure 24:**
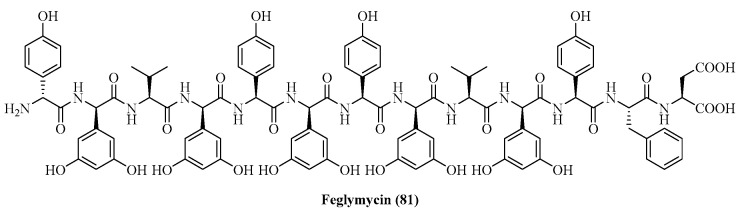
Structure of feglymycin (**81**) targeting MurA and C.

Süssmuth et al. identified feglymycin (**81**), isolated from Streptomyces strains, as an active inhibitor of both MurA and MurC [169]. Feglymycin, a 13-mer peptide, exhibited activity with an IC_50_ in the range of μM for MurA and in the range of mM for MurC (Figure 24).

## 4. Conclusions

Over the last few decades, the peptidoglycan biosynthetic pathway has emerged as a promising and attractive target for antibacterial drug discovery. Among the various enzymes involved, the Mur ligase family has drawn attention due to its exclusive presence in bacteria (not found in human cells) and as they are essential for bacterial cell wall biosynthesis. An in-depth understanding of their structures has led to the development of powerful new antibacterial agents. While each Mur ligase can be considered a unique antibacterial target, MurC-F ligases have highly conserved amino acid regions in their active sites. This characteristic can be used in the design of promising Mur ligase multi-inhibitors. A number of inhibitors from different chemical families were developed against Mur ligases, with significant inhibitory activity. Modern techniques remain highly utilized in the design of potentially active molecules, particularly with virtual screening methods [170,171]. However, none has yet been shown to have antibacterial activity. One reason could be the difficulty for these compounds to cross the bacterial membrane and reach the cytoplasm where Mur ligases activities are located. Another possibility explaining the lack of antibacterial activity may be the complexity of the Mur ligase pathway, which is high, making it difficult for the inhibitor to reach the various sites. 

In the future, a better understanding of the protein–protein interactions of the Mur ligase pathway, combined with the consideration of factors enabling better penetration of the bacterial wall, will enable the design of Mur ligase inhibitors with proven antibacterial activity.
